# The Dangers of Acetanilid

**Published:** 1904-10

**Authors:** 


### THE DANGERS OF ACETANILID.

The following editorial is a very timely caution of an evil that has assumed a startling prominence among drug-takers. It should be noted as a danger which can be prevented at present:

"There has recently been considerable discussion among statisticians and sanitarians both in this country and in England as to the cause of the increase which has been apparent during the last three or four years in the number of sudden deaths from heart disease.

"It has been suggested, and with considerable reasonableness, that many of these deaths were due to the increased use of acetanilid by large numbers of the people. There are two chief methods in which acetanilid is obtained by people. One is in the form of headache powders, which every druggist is aware are consumed in enormous quantities by the people of every class. These powders are extensively advertised and freely bought over the drug-store counter, and are also sold in many large department stores. They all contain acetanilid, a powerful cardiac depressant, which causes destruction of the blood corpuscles and transforms the hemoglobin into methemoglobin. This drug is unquestionably a powerful tissue poison, and, if used at all,—and it is doubtful if it is ever indicated,—should only be taken by the direction and under the observation of a physician. Phenacetin has the same antipyretic and analgesic properties as acetanilid, without its dangers, and should always be prescribed. Phenacetin, however, is much more expensive than acetanilid, and as it is a drug which has been in very general use by physicians during the last few years, and as it closely resembles in appearance and in its general properties acetanilid, it has been discovered that large numbers of druggists in New York, a nd doubtless elsewhere as well, have been in the habit of substituting the latter, charging for the more expensive drug and dispensing the cheaper. The New York Board of Health purchased through its inspectors 373 samples from as many druggists, asking in each case for phenacetin. On analysis it was discovered that out of the 373 samples 58 only were pure phenacetin, 315 were adulterated with acetanilid, and 32 samples were pure acetanilid. This was at the beginning of 1903, and the facts having been published, and the druggists warned that a second offense would mean prosecution, there was a marked diminution in the amount of acetanilid dispensed, and, oddly enough, the vital statistics for New York for 1903 showed a considerable falling off in the numbers of sudden deaths from heart disease. During the previous two years these deaths had been steadily increasing. We are well aware of the fallacy of such statistics, and do not admit that the relationship between the popular use of acetanilid and sudden death from heart disease had been demonstrated; but we do think that the matter is of sufficient importance to warn physicians, and through them their patients, of the undoubted dangers of acetanilid taken in the form of headache powders or otherwise, and to warn druggists that substitution is a crime carrying with it a heavy penalty for the offender."—(Editorial, *St. Paul Medical Journal,* March, 1904.)—JOSEPH M. AIKIN, Omaha.

